# Is it necessary to perform occupational audiometric testing at 6-months of employment?

**DOI:** 10.1016/j.bjorl.2020.12.008

**Published:** 2021-01-19

**Authors:** Vagner Antonio Rodrigues da Silva, Alexandre Caixeta Guimarães, Alexandre Scalli Mathias Duarte, Joel Lavinsky, Arthur Menino Castilho, Carlos Takahiro Chone, Agricio Nubiato Crespo

**Affiliations:** a Universidade Estadual de Campinas (Unicamp), Faculdade de Ciências Médicas (FCM), Departamento de Otorrinolaringologia, Cirurgia de Cabeça e Pescoço, Campinas, SP, Brazil; b Universidade Federal do Rio Grande do Sul (UFRGS), Departamento de Cirurgia, Porto Alegre, RS, Brazil

**Keywords:** Noise, Noise-induced hearing loss, Objective audiometry, Hearing loss

## Abstract

**Introduction:**

Current Brazilian legislation requires that all workers exposed to noise are to be given an audiogram upon hiring, after 6 months of employment (first periodic test), and annually after the first periodic test. In other countries, the regulations of hearing conservation programs do not include the requirement for audiometric testing at 6 months of employment, but only annually. There is no evidence that the periodicity adopted by Brazilian legislation is the most appropriate.

**Objective:**

The present study aimed to evaluate the first 3 occupational audiometric tests of workers exposed to noise.

**Methods:**

Historical cohort study with cross-sectional analysis. Participants were all male metallurgy workers aged up to 40 years. The first 3 audiograms of each worker were analyzed: pre-employment audiometric test, periodic audiometric test 1, and periodic audiometric test 2. For each worker, mean frequency thresholds were calculated at 3, 4, and 6 kHz in the left and right ears for each test. Statistical analysis was performed using the nonparametric Wilcoxon test.

**Results:**

A total of 988 workers were included. There was a significant difference in auditory thresholds between the pre-employment test and the 2 subsequent periodic tests for the right and left ears. There was no significant difference between Test1 and Test2 in either ear.

**Conclusion:**

Given the lack of difference between the first 2 periodic tests, we believe that they could be merged into a single test, i.e., first periodic audiometric testing could be performed at 12 months of employment without compromising workers’ health.

## Introduction

Exposure to noise is an important cause of hearing loss,^1^, ^2^ with noise-induced hearing loss (NIHL) ranking as the second most common occupational disease. Hearing conservation programs developed between 1945 and 1966, focusing on continuous exposure to high noise levels. In the United States, with the Occupational Health and Safety Act of 1970, Congress created a federal regulatory agency within the Department of Labor, the Occupational Health and Safety Administration (OSHA). In the same year, the National Institute for Occupational Safety and Health (NIOSH) was created to develop safe limits for workplace exposures.^3^, ^4^

In Brazil, in 1978, the Ministry of Labor published the “Regulatory Standard nº 15”, which establishes the safe limits for noise exposure — requirements that remain in place today. In 1998, the National Noise and Hearing Conservation Committee regulated work-related NIHL.^5^, ^6^ Current legislation, through Ordinance nº 19 of the Ministry of Labor and Employment, requires that all workers exposed to noise are given an audiogram upon hiring, after 6 months of employment (first periodic test), annually after the first periodic test, and on work termination.^5^, ^7^ In other countries, the regulations of hearing conservation programs do not include the requirement for audiometric testing at 6 months of employment, but only annually.^8^, ^9^, ^10^

There is no evidence that the periodicity adopted by Brazilian legislation is the most appropriate. The optimal time interval to perform the first periodic audiometric test remains unknown.

The present study aimed to evaluate the first 3 occupational audiometric tests of workers exposed to noise.

## Methods

This historical cohort study with cross-sectional analysis was approved by the Institutional Review Board (protocol nº 0810.0.146.000-11).

Data from audiometric tests performed between January 1998 and January 2018 were obtained from a Brazilian metallurgy company that had implemented hearing conservation programs according to Bulletin nº 6 of the National Noise and Hearing Conservation Committee.^7^ The variation in the sound pressure level in each company between exposed and non-exposed workers was not statistically significant. Workers exposed to ≥ 85 dB sound pressure level for at least 8 h/day, who were provided with hearing protection devices (earplugs) by the company as required by law, were recruited for participation in the study.

The first 3 audiograms of each worker were analyzed: pre-employment audiometric test, periodic audiometric test 1, and periodic audiometric test 2. Pre-employment test was defined as the worker’s first audiogram upon hiring but before starting to work. The periodic tests are hereafter referred to as Test1 (periodic test 1) and Test2 (periodic test 2).

Inclusion criteria: (1) male; (2) metallurgy workers; (3) aged up to 40 years, 11 months, and 364 days on the date of pre-employment audiometric testing; (3) 3 audiometric tests (pre-employment, Test1 and Test2); (4) 14 h of hearing rest prior to each test; (5) normal hearing (thresholds ≤25 dB HL for octave frequencies from 0.25 to 8 kHz) at the time of pre-employment audiometric testing.

Exclusion criteria: (1) female; (2) workers from other industrial sectors; (3) workers non-exposed to noise (≤80 dB sound pressure for at least 8 h daily); (4) incomplete audiometric data (absence of frequency thresholds at 3, 4 and 6 kHz bilaterally); (5) conductive hearing loss; (6) sensorineural hearing loss; (7) otologic disease (endolymphatic hydrops, otosclerosis, chronic otitis media); (8) tests performed for change of position, return to work, or work termination; (9) presence of chronic diseases (hypertension, diabetes mellitus, autoimmune diseases, infectious diseases, or immunodeficiencies), (10) complaints of tinnitus or dizziness; (11) workers had performed Test1 after 12 months and 364 days or before 5 months and 29 days of the pre-employment test and, (12) workers had performed Test2 after 24 months and 364 days of the pre-employment test or before 5 months and 29 days of Test1.

The audiometric tests were performed in specialized occupational medicine centers. Pure-tone audiometry was performed at a specialized center with experience in audiometric testing procedures for occupational noise exposure. Prior to audiometry, all ears were examined using a Heine otoscope. Three audiometers calibrated according to the International Organization for Standardization (ISO) 389/64 and American National Standards Institute (ANSI) S3.6/69 standards, were used for the testing procedures: Madsen Midimate 622 (GN Otometrics, Taastrup, Denmark); Interacoustics AD 29 (Interacoustics, Assens, Denmark); and LO-250 (Acústica Orlandi, Bauru, SP, Brazil).

All tests were performed in audiometric booths, with ambient noise levels in accordance with ANSI S3.1-1991, according to the following parameters^7^: air conduction at the frequencies of 0.25, 0.5, 1, 2, 3, 4, 5, 6 and 8 kHz; bone-conduction threshold testing at 0.5, 1, 2 and 4 kHz if the airway thresholds were altered; and speech recognition threshold and speech intelligibility index.

For each worker, mean frequency thresholds were calculated at 3, 4 and 6 kHz in the left and right ears for each test. The frequencies of 0.25–2 kHz and 8 kHz were not considered in the statistical analysis. Statistical analysis was performed using the nonparametric Wilcoxon test. Results were considered statistically significant at p < 0.05. Data were analyzed using R software.

## Results

A total of 988 workers were included, resulting in 2964 tests analyzed. Based on the date of pre-employment audiometric testing, mean age was 23.8 years; 82.3% were aged between 21 and 30 years.

The mean time elapsed between pre-employment test and Test1 was 7.46 months. Test2 was performed at a mean of 18.20 months after pre-employment audiometric testing. On average, 10.74 months elapsed between Test1 and Test2.

Table 1 shows the results for the mean 3, 4 and 6 kHz-frequency thresholds.

**Table 1:** Results for the mean 3, 4 and 6 kHz frequency threshold in the first 3 audiometric tests, stratified by laterality.

Test	Right ear		Left ear	
	Mean	SD	Mean	SD
Pre-employment	9.03	6.42	9.98	6.73
Test1	10.55	6.31	10.99	6.44
Test2	10.71	5.98	11.08	6.07

SD, standard deviation; Pre-employment, pre-employment audiometric test; Test1, periodic audiometric test 1; Test2, periodic audiometric test 2.

Table 2 shows a comparison of the mean 3, 4 and 6 kHz-frequency thresholds between tests. There was a significant difference in auditory thresholds between the pre-employment test and the 2 subsequent periodic tests for the right and left ears. There was no significant difference between Test1 and Test2 in either ear.

**Table 2:** Comparison of audiometric tests according to the mean results for the 3, 4 and 6 kHz frequencies, stratified by laterality.

Ear	Pre-emp vs. Test1	Pre-emp vs. Test2	Test1 vs. Test2
Right	*p* = 0.008	*p* = 0.000	*p* = 0.064
Left	*p* = 0.003	*p* = 0.000	*p* = 0.072

Pre-emp, pre-employment audiometric test; Test1, periodic audiometric test 1; Test2, periodic audiometric test 2.

## Discussion

Periodic audiometric testing is considered a good method to assess hearing conservation measures. Worsening of workers’ audiometric thresholds over time may indicate that hearing conservation measures adopted by the company have been ineffective. However, the pathogenesis of NIHL is still poorly understood. Exposure to noise affects the peripheral and central auditory systems, even before changes are evident on audiometric testing. Changes observed in the auditory brainstem response of young adults with normal audiograms exposed to noise suggest that there is central auditory system demyelination, but not cochlear synaptopathy.^11^

The present study evaluated workers with normal baseline audiograms in their first 2 years of exposure to noise. The frequencies of 3–6 kHz, which are early affected by noise,^2^, ^12^, ^13^ were analyzed in this study. The frequencies of 0.25–2 kHz and 8 kHz, however, were not considered in the statistical analysis because they are only later affected in noise-exposed patients.^1^, ^5^, ^14^, ^15^ Authors such as Rabinowitz et al.,^16^ Coles et al.,^12^ and Kirchner et al.^13^ recommend that occupational physicians and otolaryngologists should give special attention to the frequencies of 3, 4 and 6 kHz in workers exposed to noise.

Aging and comorbidities can worsen hearing thresholds regardless of exposure to noise.^1^ For this reason, we excluded from the study workers aged >40 years on the date of pre-employment audiometric testing as well as workers with diabetes, hypertension, and autoimmune and infectious diseases. Women were not included in the study because, in the metallurgy industry, they are mostly employed in the administrative and human resources departments, where there is no exposure to noise.

Government regulations require the use of hearing protection devices in noisy environments.^4^ However, the company under study could not provide statistical data to confirm proper use of hearing protection by all workers exposed to noise. Despite the evidence of a worsening of audiometric thresholds between pre-employment audiometric testing and the second periodic test, numerically, the difference was not relevant. Therefore, we can infer that hearing conservation measures implemented in the company under study have been effective.

In Brazil, mandatory medical examinations are performed annually in workers exposed to hazards, except in special situations (Ordinance nº 24, 1994).^17^ The legal requirement for first audiometric testing to occur at 6 months of employment, and then annually thereafter, for workers exposed to hazardous noise levels (Ordinance nº 19, 1998)^18^ makes medical examinations no longer coincide with audiometric testing. In most workers evaluated in the present study, the first periodic audiometric test was not performed at 6 months of employment, as required by law.^7^ On average, this test was performed at 7.46 months of employment. Of 988 workers analyzed, only 401 (40.58%) underwent the first periodic test at 6 months of employment. The second periodic test was performed, on average, at 10.74 months after the first periodic test.

The noise-exposed workers in the present study showed a statistically significant difference between the results of pre-employment audiometric testing and those of the first 2 periodic tests. However, there was no significant difference between the 2 periodic tests. The lack of difference between the 2 periodic tests may be explained by the possible triggering of a self-protection mechanism after the beginning of the exposure, which, due to the use of hearing protection and engineering measures, was not very intense. This protective effect is similar to that triggered by a non-ototoxic dose of aminoglycoside administered before the ototoxic dose of the same antibiotic.^19^, ^20^

Table 1 shows asymmetry between the right and left ears since the pre-employment test, with worsening of the thresholds in the left ear. Nageris et al.^21^ and Fernandes and Fernandes^22^ showed asymmetry between the ears and, although in some workers the right ear had higher hearing thresholds than the left ear, in most cases the cause of asymmetry was the increased thresholds in the left ear. One possible explanation for the variation in noise susceptibility is a variation in the functional activity of the medial olivocochlear efferent system. It has been shown that the medial olivocochlear efferent system is stronger in the right ear than in the left in humans.^23^ Neuropsychological studies have demonstrated that speech perception is lateralized in the central nervous system, with involvement of the left upper and middle temporal gyri. Temporary changes in hearing thresholds after binaural exposure are greater in the left ear than in the right ear.^24^

Hearing is considered to have worsened if there is an increase of more than 10 dB in the mean results for 3 consecutive frequencies, such as 3, 4 and 6 kHz, in subsequent audiograms. In the present study, the difference was on average less than 10 dB between subsequent tests. Although it is not possible to state that hearing worsened in the workers, we may assume that initial exposure to noise is probably causing some hearing damage despite the hearing conservation measures imposed by current legislation.

## Conclusion

Metallurgy workers exposed to noise had a significant worsening of mean audiometric thresholds at 3, 4 and 6 kHz between pre-employment audiometric testing and the first 2 periodic audiometric tests. However, these thresholds did not differ significantly between the first and second periodic tests. Therefore, given the lack of difference between the first 2 periodic tests, we believe that they could be merged into a single test, i.e., first periodic audiometric testing could be performed at 12-months of employment, decreasing the companies’ costs, without compromising workers’ health, which is in accordance with worldwide regulations.

## Conflicts of interest

The authors declare no conflicts of interest.
